# Hyaluronic Acid-Mediated Phenolic Compound Nanodelivery for Cancer Therapy

**DOI:** 10.3390/pharmaceutics15061751

**Published:** 2023-06-16

**Authors:** Simona Serini, Sonia Trombino, Federica Curcio, Roberta Sole, Roberta Cassano, Gabriella Calviello

**Affiliations:** 1 Department of Translational Medicine and Surgery, Section of General Pathology, School of Medicine and Surgery, Università Cattolica del Sacro Cuore, Largo F. Vito, 00168 Rome, Italy; simona.serini@unicatt.it; 2 Fondazione Policlinico Universitario A. Gemelli IRCCS, Largo F. Vito, 00168 Rome, Italy; 3 Department of Pharmacy, Health and Nutritional Sciences, University of Calabria, Arcavacata di Rende, 87036 Cosenza, Italy; sonia.trombino@unical.it (S.T.); federica.curcio@unical.it (F.C.); roberta.sole@unical.it (R.S.)

**Keywords:** cancer, chemotherapy drugs, hyaluronic acid, nanoformulations, polyphenols, targeted delivery

## Abstract

Phenolic compounds are bioactive phytochemicals showing a wide range of pharmacological activities, including anti-inflammatory, antioxidant, immunomodulatory, and anticancer effects. Moreover, they are associated with fewer side effects compared to most currently used antitumor drugs. Combinations of phenolic compounds with commonly used drugs have been largely studied as an approach aimed at enhancing the efficacy of anticancer drugs and reducing their deleterious systemic effects. In addition, some of these compounds are reported to reduce tumor cell drug resistance by modulating different signaling pathways. However, often, their application is limited due to their chemical instability, low water solubility, or scarce bioavailability. Nanoformulations, including polyphenols in combination or not with anticancer drugs, represent a suitable strategy to enhance their stability and bioavailability and, thus, improve their therapeutic activity. In recent years, the development of hyaluronic acid-based systems for specific drug delivery to cancer cells has represented a pursued therapeutic strategy. This is related to the fact that this natural polysaccharide binds to the CD44 receptor that is overexpressed in most solid cancers, thus allowing its efficient internalization in tumor cells. Moreover, it is characterized by high biodegradability, biocompatibility, and low toxicity. Here, we will focus on and critically analyze the results obtained in recent studies regarding the use of hyaluronic acid for the targeted delivery of bioactive phenolic compounds to cancer cells of different origins, alone or in combination with drugs.

## 1. Introduction

According to recent estimates, cancer represents the first or second highest cause of death in most countries around the world, with nearly 10 million deaths globally reported in 2020 (WHO). Moreover, a dramatic increase in the number of new cancer cases is expected over the next decades, with an increase of 47% from 2020 to 2040 [1]. The high prevalence of cancer and its increase is due to the aging/growth of the population and to risk factors that continue to represent the predominant cause of cancer in many countries (such as tobacco smoking in Western countries or viral infection in developed countries) or that are escalating all over the world (such as overnutrition and overweight) [2]. It is noteworthy that the same risk factors associated with cancer (chronic infections, tobacco smoking and chronic alcohol abuse, chronic pollutant inhalation, overnutrition and obesity, and autoimmunity) are also related to chronic inflammation [3]. This is in keeping with the close connection existing between chronic inflammation and cancer since, on the one hand, chronic inflammation may precede and be strictly related to both tumorigenesis and cancer progression. On the other hand, the tumor itself may intrinsically induce an inflammatory response, and, in the long term, the recruited inflammatory cells can also create an immunosuppressive environment able to facilitate the progression of cancer [4]. This explains why factors able to negatively regulate the inflammatory response are considered important tools in the prevention of cancer and may also exert important roles in the treatment of cancer. Accordingly, in this review, we are focusing on phenolic compounds (PheCs), natural dietary components, and nutraceuticals, which have been recognized as powerful anti-inflammatory and antioxidant agents and largely proposed for the prevention of cancer and as adjuvants in anticancer therapy [5]. We will examine the possibilities that have been recently explored (and published within the last 5 years: 2018–2023) for more innovative use of them in cancer therapy. In particular, we will critically analyze the results of papers that have investigated the delivery of PheCs through hyaluronic acid (HA)-based systems with the aim of improving chemotherapy.

### 1.1. Why Should Chemotherapy Be Improved?

Chemotherapy represents one of the possible conventional strategies for treating cancer, together with surgery and radiotherapy, while remarkable results have been achieved in the last two decades thanks to the use of alternative and innovative approaches, such as molecular targeted therapy and immunotherapy. On these bases, investigating and trying to ameliorate chemotherapy could be considered quite obsolete. Particularly, immunotherapy represents a change in paradigm in cancer therapy since, in this case, the antineoplastic agents, instead of directly contrasting cancer cells, have the potential to activate the patient’s immune system to become able to attack cancer cells powerfully and successfully. However, it should be underlined that the drawbacks existing for the conventional therapeutic approaches, such as low response rate, dangerous side effects, development of resistance, immunosuppression, and high recurrence rate chemotherapy, are also reported for the most innovative therapeutic strategies, and new routes are being investigated to overcome these problems [6,7,8].

Therefore, in this scenario, in concomitance with the tremendous upsurge of interest in immunotherapy, comparable efforts are currently being expended to innovate chemotherapy and make it more efficient and tolerable for patients. They are crucial since, even though large subsets of patients can now be cured by using molecular targeted therapy and immunotherapy, chemotherapy, accompanied by surgery or not, remains the first line and the gold standard for the eradication of most tumors in the majority of patients.

Moreover, it has recently become clear that the therapeutic potential of chemotherapeutic agents should not be only restricted to their capacity to exert direct cytotoxic effects on cancer cells. In fact, it has been observed that chemotherapy can powerfully reinforce the natural but unsuccessful immune response against tumors and also potentiate and prolong the benefit of immunotherapy when they are administered in combination [9,10]. This represents an important advancement in cancer therapy, which is in part related to the recent revisitation of cancer chemotherapeutic drugs as inducers of immunogenic cell death (ICD). This is a process that allows tumor cells to become immunogenic as they undergo a form of programmed cell death (PCD) under the effect of an external stimulus, making them able to induce a powerful and long-lasting antitumor immune response [11]. Interestingly, it has been reported that ICD is in strict relationship with several immunogenic forms of PCD, such as necroptosis, ferroptosis, pyroptosis, or autophagy [11]. These forms of PCD are characterized by the cellular discharge/secretion of damage-associated molecular patterns (DAMPs, including calreticulin, ATP, and several heat shock proteins), as well as other pro-inflammatory factors such as cytokines and chemokines, which, once released into the extracellular milieu, induce the activation of the innate immune and adaptive immune system [12]. In particular, both DAMPs, by binding to the pattern recognition receptors (PRRs) expressed in dendritic cells (DCs), and cytokines promote the maturation of DCs and their appropriate presentation of tumor antigens to T cells, which ultimately activate an immune response leading to ICD. This, in turn, promotes PCD, thus allowing a sustained anti-tumoral immunity efficacy [11]. It has been observed that even apoptosis, which has been generally considered a non-immunogenic form of death not able to induce ICD, may switch and become immunogenic if it is caused and/or accompanied by endoplasmic reticulum (ER) stress [13]. Interestingly, several anticancer treatments, including chemotherapy with anthracyclines, have been seen to have the potential to produce ROS, which is closely related to the ER stress induction in tumor cells, and thus convert apoptosis into an immunogenic form of death [14].

### 1.2. Strategies for Innovating Anticancer Chemotherapy

The different strategies pursued in the effort to innovate chemotherapy and make it more efficient and tolerable for patients are mainly aimed to: (a) enhance the water solubility of low-solubility or insoluble drugs, thus increasing their bioavailability; (b) protect the drugs from destruction and deactivation during their transport to the tumor tissues; (c) specifically deliver the drug to the target cells while sparing normal tissues from drug-induced damages, thus avoiding undesired systemic effects.

Among the new approaches used to make chemotherapy more tissue specific, efficient, or less threatening for patients, there is the conjugation of drugs with a variety of carriers. Particularly, polymeric water-soluble macromolecules covalently linked with hydrophobic drugs allow them to enhance their water solubility and increase their bioavailability. In fact, depending on the characteristic of the carrier, they may protect the drugs from possible oxidative and enzymatic attack, as well as from their reticuloendothelial system cell uptake while in circulation, and specifically target them to the pathological site [15].

Another possibility is the inclusion of chemotherapeutic agents in inorganic (such as metal nanoparticles, carbon-based nanoparticles (NPs)) or organic nanostructured materials (such as liposomes, micelles, or NPs), as well as in hydrogels or microparticles [15]. Among the others, NPs, for their physical and chemical properties, seem particularly suitable carriers, having an ultra-fine size and sub-cellular function [16]. In particular, the design of nanosystems for anticancer drug delivery has attracted considerable attention since they show the potential to target tumor cells both passively and actively [17]. The passive targeting is related to the known permeability and retention effect (EPR) of the tumor endothelium, which, while allowing small drugs to cross it in both directions, induces the nanosystems to passively accumulate in the interstitium surrounding cancer cells and reach them with higher efficiency [18]. On the other hand, the active tumor-targeted delivery has allowed further remarkable advances in making the treatments more tumor-specific. It consists of the use of NPs incorporating on their surface a variety of compounds or antibodies, which drive specifically the NPs and the loaded drugs toward the pathological sites while preserving normal tissues [17]. The hydrogels [19] are also considered smart drug carriers for cancer for their high ability to bind and carry hydrophobic drugs to their destination while protecting them from enzymatic degradation.

Further approaches have also been explored to reinforce the effect of anticancer chemotherapy, including the design of systems able to simultaneously carry antineoplastic drugs and bioactive natural compounds known to possess antioxidant, anti-inflammatory, and anticancer activities themselves, such as PheCs, omega-3 fatty acids, metabolites derived from garlic or *Brassicaceae*, or many others [20,21,22,23]. Interestingly, some natural products have also been proven to promote ICD by themselves, independently from the effects of antineoplastic drugs [24,25,26]. Therefore, currently, the combined delivery of chemotherapeutic drugs with natural bioactive products, in combination or not with immunotherapy, appears to be an exciting new area of research. The delivery of some natural products independently from any chemotherapeutic treatment is also often investigated since it has been shown that, for their properties, these products are able to exert powerful antineoplastic activities also by themselves.

## 2. PheCs Included in Drug Nanodelivery Systems for Tumor Targeting

**Figure 1:**
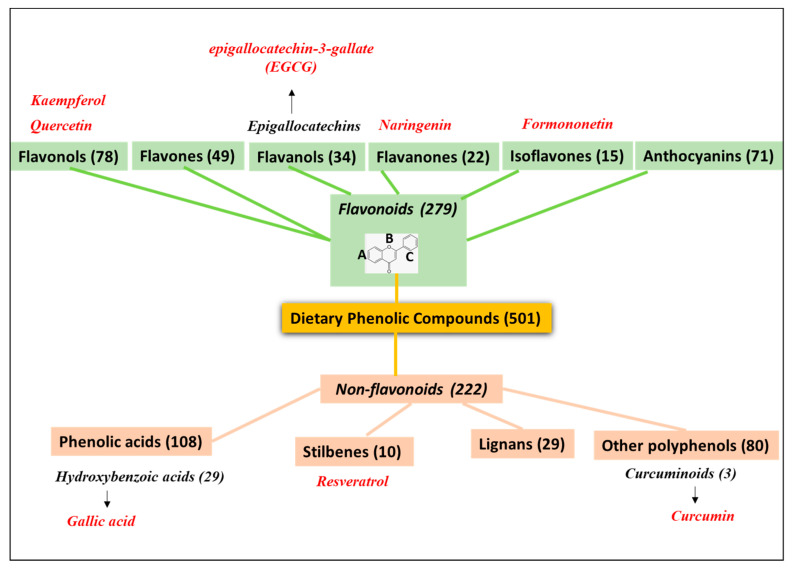
Classes of natural dietary phenolic compounds (PheCs) according to the version 3.6 of the Phenol-Explorer database regarding the polyphenol content in foods (http://phenol-explorer.eu/compounds, accessed on 24 March 2023).

Among the bioactive natural products investigated for reinforcing the anticancer effects of chemotherapy treatments and recently included in a series of nanodelivery systems developed for tumor targeting, we will focus here on PheCs, a large group of natural compounds extracted by plants. They are characterized by the presence of at least one phenol ring in their moiety and are well known for their powerful anti-inflammatory, antioxidant, and anticancer properties [27]. In particular, plenty of results obtained both in in vitro and in vivo have supported the powerful effects that a series of PheCs may exert against the development and progression of a variety of cancers [28]. The antineoplastic activities have been described for compounds belonging to all the classes of PheCs [29,30]. In fact, this represents the most numerous group among the phytochemicals present in fruits, vegetables, and other foods [31], and, according to version 3.6 of the Phenol-Explorer database regarding the polyphenol content in foods [32], there are over 500 PheCs present in our diet that can be classified in different classes. Even though the total number of identified PheCs is much larger compared to those found in our diet [33], for the sake of simplicity, we will follow the one reported in the Phenol-Explorer database. The most numerous class is that of *flavonoids*, which, according to this classification, includes 279 compounds present in our diet, divided into the sub-classes of anthocyanins, chalcons, dihydrochalchones, dihydroflavonols, flavanols, flavanones, flavones, flavonols, and isoflavonoids (Figure 1). *Flavonoids* are polyphenolic compounds grouped in a single class on the basis of their common 15-carbon skeleton formed by two benzene rings (A and C, see Figure 1) bound together by a heterocyclic pyrane ring (B in Figure 1). They are ubiquitously found in plants, where they play a role in response to microorganism infections [33]. Their recognized antioxidant and free radical scavenging activity is mediated by the hydroxyl groups bound to their phenolic rings. These hydroxyls are also able to chelate metal ions and, thus, hamper the metal-catalyzed generation of reactive oxygen substances (ROS) and their oxidant activity. According to our research of the literature, the *flavonoids* that have been included in HA-coated delivery nanosystems investigated against cancer in the last 5 years are the two *flavonols:* kaempferol [34] and quercetin (QU) [35,36,37,38,39,40,41], the *flavanol* epigallocatechin-3-gallate (EGCG) [42,43,44,45,46,47] the *flavanone* naringenin [48], the *isoflavone* formononetin [49], and a mix of *anthocyanins* extracted from corn [50].

Between brackets are reported the number of compounds belonging to each class or subclass according to this classification. In red are the PheCs that have been included in HA-coated delivery nanosystems and investigated in the last 5 years: 2018–2023.

For the sake of simplicity, we have included in one single class (named *non-flavonoids*) all the other PheCs non-belonging to the class of *flavonoids* (see Figure 1). The major *non-flavonoid* subclass for numerosity is that of the phenolic acids containing over 100 compounds present in our diet (Figure 1). They include in their moiety a single phenolic ring with an organic carboxylic acid bound to it. They have been further divided into different subgroups, among which hydroxybenzoic acid and hydroxycinnamic acid groups are the most prevalent. Phenolic acids are widespread in foods and contribute to their organoleptic characteristics (color, flavor, astringency, and harshness) as well as to their nutritional properties. In fact, similarly to what was observed for the compounds belonging to the class of polyphenols *flavonoids*, they exert antioxidant activities and have been observed to protect from neurodegeneration [51,52]. Moreover, they may induce many other health benefits, including the prevention of neoplastic, metabolic, and cardiovascular diseases [53,54]. The only phenolic acid included so far in an HA-coated delivery system for the targeting of cancer is gallic acid [55], which belongs to the class of hydroxybenzoic acids. Other phenolic acids (dihydrocaffeic acid, ellagic acid), however, were recently included in HA-based NPs for preventing degenerative or inflammatory conditions known to be pro-carcinogenic, such as those induced by UVB radiations [56] or associated with chronic inflammatory bowel diseases [57]. Moreover, gallic acid was also included in an HA-based immunosuppressive hydrogel for possible applications in wound healing and tissue regeneration [58].

Then, according to the same classification, among the dietary PheCs have also been included the 2 less-abundant groups of the *Stilbenes* and *Lignans* (Figure 1), and finally, under the name of *Other Polyphenols*, a series of 80 compounds among which there is the *curcuminoid* curcumin (CUR), which has been largely reported to have a prominent antioxidant and anticancer activity [59] (Figure 1). In particular, *stilbene* resveratrol [60,61,62,63] and *curcuminoid* curcumin [64,65,66,67,68,69,70,71,72,73,74,75] are the *non-flavonoids* that have been so far included in HA-coated delivery systems for possible cancer targeting.

For their presence in our diet, PheCs are generally considered safe and usable, with no trouble for the design of new drugs. However, it should be underlined that PheCs may interact and interfere with many currently used conventional drugs, making their use not completely devoid of risks for some classes of patients [76]. In particular, Gómez-Garduño et al. [76] reported that the interactions of PheCs with drugs often involve cytochrome CYP3A enzymes and P-gp transporters and are mediated through the regulation of gene expression or inhibitory effects on functional proteins that, ultimately, may modify plasma concentrations of drugs. These interactions are presumably related to the distribution of phytochemicals in circulation and, from there, to all our organs and tissues. This underscores how useful and safe it may be for therapeutic scopes, introducing these bioactive natural compounds not in a free form but included in drug-delivery systems, which, besides protecting them from unwanted chemical interactions, may mainly and specifically transport them to tissues where pathologic processes are ongoing.

As indicated above, we are here restricting our critical analysis to papers focused on the improvement of anticancer chemotherapy obtained through the inclusion of PheCs in delivery systems. However, we are excluding those papers focusing on nanoformulations that include PheCs not on the basis of their recognized antitumor effects but just for their ability to bind firmly to other molecules and form an envelope able to include and protect and/or more easily release the anticancer drugs in the tumor environment. In fact, for instance, due to their ability to self-assemble to metals, PheCs can form the metal-phenolic networks (MPNs) that have recently received considerable attention for their possible biological applications as coating materials suitable not only for drug delivery but also for the improvement of bioimaging and encapsulation of cells [77]. These coatings are quickly and easily obtained by mixing a PheC and a metal cation in the presence of a substrate. Therefore, for these applications, PheCs are used only since they contain aromatic rings with hydroxyl groups, which may serve as multivalent chelation sites able to interact with metal ions, thus giving the possibility of forming a coating network on a variety of substrates independently from their features, surface charge, or shape [78]. In particular, PheCs that contain dihydroxyphenyl (cathecol) or trihydroxiphenyl (galloyl) groups can be used for interface engineering and particle development, thanks to the possibility of establishing through them covalent and non-covalent interactions needed for the assembly of PheC-based materials [78]. On the other hand, some PheCs, such as anthocyanins, have also been used as promising nanovectors for chemotherapic drugs, and not on the basis of their known powerful anticancer properties [79]. For instance, in a recent study by Xiong et al. [50], the *flavonoids* anthocyanins were used as vectors in NPs designed for the delivery of the antineoplastic drug doxorubicin to colon cancer cells. In this case, the anthocyanins were one of the components of the hydrophobic core of the nanosystem, and for their property of being ROS-responsive, they had the potential to break the covalent bonds to allow the preferential release of the covalently linked drug in the ROS-enriched tumor microenvironment.

## 3. Hyaluronic Acid: An Efficient Carrier for the Specific Delivery of Antineoplastic Drugs and Natural Bioactive Products

As indicated above, we are here concentrating on the results of recently published papers focused on the hyaluronic acid (HA)-based delivery of PheCs to tumor tissues. Multiple properties of HA make this polysaccharide a particularly efficient platform for the specific delivery of antineoplastic drugs and natural bioactive anticancer therapeutics. In fact, it is constituted by repeating units of the disaccharide D-glucuronic acid and N-acetyl-D-glucosamine, and it is naturally synthesized by our body. It represents a main constituent of the extracellular matrix and a ubiquitous component of our tissues [80], where it exerts crucial roles in some cellular processes, such as growth, differentiation, and migration [81,82]. Altogether, this is enough to explain its high biocompatibility and lack of toxicity or immunogenicity. These features are crucial when considering its possible drug-delivery application. In fact, most synthetic polymers and inorganic materials that have been so far explored for this use have limitations, such as non-negligible in vivo toxicity [64]. However, additional reasons make HA particularly attractive as a drug-delivery carrier, such as its biodegradability, as HA is degraded by multiple enzymes in the microenvironment of tumors, thus allowing easy release [83]. Moreover, other critical reasons for its use in nanoformulations are its ability to be easily chemically modifiable and its hydrophilic properties, which make it particularly apt to transport hydrophobic drugs [84]. In addition, it shows a high affinity for the cluster of differentiation-44 (CD44), a transmembrane glycoprotein that, besides participating in physiological processes, including cellular adhesion and migration in inflammation and repair [85], has been found to be overexpressed on the surface of a variety of cancer cells, particularly in solid tumors such as breast, cervical, and prostate cancer and glioblastoma [86]. This receptor was found to be significantly upregulated in cancer stem and metastasizing cells, representing a biomarker of cancer cell stemness [87] or epithelial–mesenchymal transition [88]. In fact, it has been involved in the survival of stem cells, as well as in invasion, metastasis, neoangiogenesis, and drug resistance and recurrence [86,89,90,91]. This means that a delivery system coated with HA has the potential to reach specifically the stem cells inside the primary cancer, as well as the metastases spread from it. Finally, it has been reported that CD44 could play not only the role of a “binder” for a ligand such as HA but also that of an “internalizer”, thus facilitating not only the specific targeting of cancer cells by HA-coated drug systems but also improving their endocytosis and accumulation inside the target cells, thus enhancing the antitumor efficacy of the delivered drugs [92]. Altogether, this means that the HA-containing drug-delivery systems, due to their ability to bind with high affinity to the CD44 receptor on cancer cells, could have the potential to overcome the remarkable systemic side effects usually induced by most antineoplastic chemotherapeutic drugs characterized by a very poor tissue specificity. A very recent comprehensive review summarized the possible impact on cancer therapy of different HA-based nanocarriers designed for the encapsulation of antineoplastic drugs [93].

There are different forms of HA-based delivery systems that have been designed and used for a more specific, efficient, and safe cancer therapy. The simplest and largely used way to specifically carry an anticancer agent in combination with HA is to conjugate it to the HA moiety itself. For instance, it has been largely shown that conjugation of HA with several anticancer drugs (such as paclitaxel, taxol, CPT11) can significantly increase the growth-inhibiting effect of the drugs in CD44-overexpressing cells, including breast, colorectal, esophageal, gastric, lung, and ovarian cancer cells (for a review, see [91]). Moreover, drug conjugation with HA allows for enhancing the water solubility of scarcely soluble drugs.

Moreover, HA has been used to form both inorganic and organic nanostructures. Among the last, there are amphiphilic polysaccharide polymeric micelles, where HA can function both as a hydrophilic polymer forming the external part and as a ligand for targeting CD44 that is overexpressed on many cancer cells [15]. Liposomes, which can encapsulate both lipophilic drugs in the lipid bilayer and hydrophilic drugs in the aqueous core, have also been often decorated with HA to make the targeting cancer specific. Similarly, HA-decorated nanoparticles of different kinds, as well as HA-decorated nanogels, have been extensively used for more specific delivery of chemotherapeutic drugs (for a review, see [15]). HA-based nanogels have also been designed, where HA represents a constituent of the matrix encapsulating the drug.

## 4. Newly Developed HA-Based Nanocarrier Systems Loading PheCs and Their Effects against Cancer

### 4.1. HA-Based Nanocarrier Systems Loading Flavonoids

In the last five years (2018–2023), multiple HA-based nanocarrier systems were developed for the specific transport of PheCs to cancer tissues (Table 1). As far as *flavonoids* are concerned, the *flavonol* QU is the most used, in agreement with the large number of results previously obtained on the anticancer effect of this PheC [94,95]. Moreover, many QU-enclosing drug-delivery nanosystems have been developed so far with the aim of enhancing the anticancer properties of other drugs or of QU itself [96]. Here, we have specifically focused on those HA-containing nanosystems that carry QU to the tumors, which makes the delivery highly specific for cancer cells, and have analyzed the results obtained in relation to the tumor cells targeted. In the last five years, six reports have used the strategy of enclosing QU in HA-based nanocarriers for transporting it (alone or with other drugs) to cancer cells (Table 1). Among these works, most of them [35,38,39,40] investigated the anticancer effects of these QU-encapsulating nanosystems on breast cancer (BCa) cells. In Xiong et al.’s work [39], the QU-encapsulating nanosystem was constructed only by mixing QU and HA. Thus, in this case, QU was the only anticancer agent delivered to the very aggressive 4T1 triple-negative breast cancer (TNBC) mouse cells grown in vitro or transplanted in syngeneic immunocompetent Balbc/mice.

The nanoformulation elicited greater anticancer effects compared to those observed when QU was administered in the free form both in vitro and in vivo. Interestingly, the authors also investigated the effect of this nanoformulation on HepG2 hepatocarcinoma cells, which express the CD44 HA ligand at a much lower level than 4T1 TNBC cells. Accordingly, both the uptake and the inhibitory effect on cancer cell growth of the HA-based QU-containing nanosystem were more pronounced in the TNBC cells, underscoring that its potential use could be useful especially in cancer cells expressing high levels of the CD44 ligand [39]. In another work, QU was enclosed in nanomicelles constituted by HA conjugated to D α-Tocopheryl polyethylene glycol 1000 succinate (TPGS), while the chemotherapeutic drug doxorubicin (DOX) was enclosed separately in an identical carrier [40], and their simultaneous administration was tested on BCa cells. TPGS is a hydrosoluble succinate ester of vitamin E, which, besides representing an optimal constituent for nanocarriers delivering liposoluble ingredients, is also known to block the ATP binding sites of P-gp ATPase, thus inhibiting its ATPase activity. This means that the nanomicelles constituted by HA conjugated to TPGS not only have the potential to specifically carry the enclosed drugs to tumor cells but also to inhibit the drug resistance that often arises in them since P-gp is one of the most representative ATP-binding cassette transporters involved in the efflux of anticancer drugs. In this case, the authors used human TNBC MDA-MB-231-MDR1 cells, which are resistant to anthracyclines such as DOX, and tested them in vitro or after transplantation in nude mice. The use of HA for decorating NPs to be delivered to this TNBC cell line appears to be very appropriate. In fact, it was observed that 85% of the TNBC MDA-MB-231 cell subpopulation expressed very high levels of CD44 compared to the very low level of CD44 expression observed in normal breast epithelial cells [97]. The HA-based nanomicelles encapsulating QU or DOX were administered both in vivo and in vitro, and they elicited the best anticancer effects with respect to either free DOX or QU. The combination also induced the maximal reducing effect on the expression of the transporter P-gp. In the remaining studies [35,38], QU and the antineoplastic drug paclitaxel (PTX) were co-delivered in a single NP enclosing both the compounds. PTX is often used successfully as the first-line chemotherapeutic agent against BCa and various other kinds of carcinomas. However, PTX induces a series of deleterious side effects, and cancer resistance often arises to the drug that may strongly reduce its anticancer efficiency. Guo et al. [35] constructed hybrid polymeric NPs formed by redox-sensitive PTX/polyethyleneimine–tocopherol hydrogen succinate–dithioglycollic acid polymeric NPs and pH-sensitive hyaluronic acid–QU conjugates. They tested their nanoformulation in vitro on MCF-7/ADR human BCa cells and MCF-7/ADR tumor 3D spheroids and on the same cells transplanted in the breast of nude mice. MCF7 cells are slowly growing cells expressing estrogen, progesterone, and glucocorticoid receptors, thus showing features of lower malignancy with respect to TNBC cells. In Qian et al.’s study [38], amphiphilic polyethyleneimine-tocopherol hydrogen succinate/hyaluronic acid-quercetin (PEI-TOS/HA-QU) core-shell micelles were constructed. In particular, the authors aimed to overcome the multidrug resistance and, thus, increase the antineoplastic efficacy of PTX, in view of the known inhibition exerted by QU on P-gp. As a matter of fact, when the nanomicelles were tested on MDA-MB-231-MDR1 BCa cells both in vivo and in vitro, they showed an increased therapeutic efficiency as compared to free PTX or QU administered separately. These results show that the treatment with HA-based nanoformulations ensuring the specific targeting of cancer cells and enclosing QU, accompanied or not by conventional drugs, could efficiently treat different kinds of BCa, even TNBC, which cannot take advantage of hormone or targeted therapies used for HER2-positive BCa or for those expressing hormone receptors. An analogous HA-based nanosystem, but co-delivering QU and gemcitabine, was tested against human pancreatic Mia-Pa-CA2 and PANC-1 adenocarcinoma cells [41]. Gemcitabine is a pyrimidinic antimetabolite able to inhibit DNA synthesis and particularly used for bladder, breast, lung, ovary, and pancreas cancer treatment. The HA-based nanoformulation was found to inhibit pancreatic cancer cell growth in vitro more than QU and gemcitabine administered separately and in the free form. This finding is particularly interesting since it has been found that the expression of the HA CD44 ligand is a feature of stemness in pancreatic cancer cells, which directly correlates with the worst outcome of patients [98]. This means that these HA-decorated nanoparticles could have the potential to be delivered specifically toward the tumors and, in particular, to their stem cell compartment.

Kaempferol, another *flavonol,* was recently encapsulated in a HA-modified nanostructured lipid carrier for the therapy of lung cancer [34]. The authors constructed two NPs and tested them against A549 lung carcinoma cells. Even though less known than QU, kaempferol has been previously shown to induce anti-inflammatory, antibacterial and antioxidant effects [99,100]. Moreover, it has the potential to prevent cardiovascular diseases [101] and to regulate several cell transduction pathways involved in cancer cell growth, migration, invasion, and promoting apoptosis [102,103,104,105]. The peculiarity of this work was the use and comparison of HA of different molecular weights (M.W.), M.W. 200–400 kDa and M.W. 1300–1600 kDa, to prepare two different nanoformulations containing kaempferol, since it was observed that the biological activities of HA of different M.W. may substantially vary [106]. They found that a higher proliferation-inhibiting activity was shown not only when kaempferol was administered to lung cancer cells enclosed in the nanoformulation but especially when the cells were treated with the nanosystem containing HA with the lowest M.W. The authors demonstrated that the nanosystem containing HA at the lowest M.W. exerted a greater anticancer activity in terms of inhibition of tumor cell migration and invasion, apoptosis induction, and expression of molecules involved in the EMT. These findings are interesting since they suggest that particular attention should be paid also to the molecular features of HA used, also considering that HA with different M.W. may even promote cancer [107]. In agreement with these results, low-molecular-weight HA (200 kDa) was previously shown to be able to control regular tissue homeostasis and exert antitumor effects [108,109]. These results were recently confirmed in a recent report of ours [107], where an HA-based hydrogel (with HA having a M.W. of 300 kDa) delivering cisplatin was able to exert remarkable antineoplastic effects in ovarian cancer cells in vitro and overcome their resistance to cisplatin.

Among the *flavonoids*, a series of studies was recently published where the *flavanol* epigallocatechin-3-gallate (EGCG) was enclosed in HA-based nanosystems and tested in cancer cells of different origins (breast, gastric, lung, and prostate cancer, as well as melanoma) (Table 1) [42,43,44,45,46,47]. This is related to the large amount of encouraging results previously obtained in preclinical studies focusing on the anticancer effects of EGCG against different kinds of cancers [110]. In two studies, the HA–EGCG nanoformulations were tested in human PC3 prostate cancer cells (in vitro and transplanted in nude mice), co-carried in one case with DTX [42] and in the other with curcumin (CU) [44]. One of these formulations [42] was constituted by HA conjugated to TPGS, that, as we have previously reported, was also recently used as an efficient carrier for the separate, but concomitant specific delivery of both QU and DOX to human TNBC MDA-MB-231-MDR1 cells [40]. In addition, in this case, the authors found that this nanocarrier was more efficient in inhibiting the growth of prostate cancer cells, both in vivo and in vitro. A similar formulation containing HA conjugated to TPGS was recently used by Huang et al. [46] to successfully inhibit the growth of gastric cancer cells grown both in vitro and in vivo. In this case, to make their nanoformulation containing EGCG and DOX more efficient in targeting gastric cancer cells, the authors constructed a dual-ligand-targeting NP by coating it also with fucoidan, a seaweed constituent showing a high affinity for P-selectin, an adhesion molecule overexpressed in the endothelial cells lining vessels in human cancers, which facilitates the formation of metastases [111,112]. Moreover, being a known apoptosis promoter and proliferation inhibitor [113], fucoidan could, in part, explain the greater efficiency shown by EGCG and DOX encapsulated in the NPs in promoting gastric cancer cell apoptosis and inhibiting their proliferation, compared to the EGCG–DOX free solution.

A particularly interesting and innovative strategy was pursued by Ding et al. [43] in constructing an EGCG-containing nanogel to be tested in human MDR1 MDA-MB-231 BCa cells both in vitro and in vivo. EGCG with its galloyl and catechol groups can easily and non-covalently interact with DNA and RNA, forming complexes. In this case, the nanosystem was obtained thanks to the electrostatic interaction between the biodegradable medium protamine sulfate and a siRNA, which was added with the aim of silencing connective tissue growth factor (CTGF), overexpressed in TNBC and associated with both drug resistance and the high degree of cell proliferation exhibited by this tumor. Then, the protamine–siRNA nanocomplex was allowed to self-assemble into EGCG. This nanosystem was able to increase the sensitivity of the tumor to EGCG antineoplastic action, thanks to the synergistic action exerted by the *flavanol* and the siRNA. Moreover, for the presence of highly biodegradable protamine in the kernel and HA on the surface, this nanogel could minimize the damage to normal tissues. Two other interesting, innovative approaches were recently developed by Song et al. [45] and Bao et al. [47]. The first group of authors [45] aimed to improve the effects of antineoplastic immunotherapy against melanoma and co-delivered EGCG and the immune modulator drug resiquimod by using their HA-based nanogels. In fact, EGCG was also shown to be active in inhibiting cancer cell PD-L1 expression, while resiquimod may act as an activator of antitumor immune responses by binding to the toll-like receptors placed at the surface of dendritic cells. Both the compounds were bound to cyclodestrins that, for their small size and solubility, ensured an enhanced penetration in the tumor tissue. Moreover, the nanogel also comprehended the pH-sensitive ketone cross-linker 2,2-dimethacroyloxy-1-ethoxypropane (DMAEP), which allowed the enclosed drugs to be better and more specifically released in the acidic tumor microenvironment. The results obtained showed an improved tumor accumulation and cellular uptake of the drugs, as well as an enhanced immune response, which corresponded to greater tumor suppression and mouse survival in the experiments performed in vivo. Instead, Bao et al. [47] designed EGCG-containing HA-based NPs that could co-deliver EGCG and a tumor-localizing photosensitizer (IR780) to be used in phototherapy combined with chemotherapy, possibly exerting a synergistic antineoplastic action. By testing their multiple-function nano-scale carrier platform on A549 lung carcinoma cells in vitro or the same cells growing in zebrafish embryos, they found that the NPs inhibited lung cancer cell proliferation in vitro and efficiently blocked tumor cell growth under laser irradiation, as well as invasion and metastasis in vivo.

Among the *flavonoids,* besides QU and EGCG, which were the most used, just naringenin (a *flavanone*) [48] and formononetin (an *isoflavon*) [49] were recently investigated for their possible delivery to cancer cells through HA-based nanoformulations (Table 1). In both cases, the treatment with the NPs inhibited the viability of cancer cells in vitro and reduced their growth when transplanted into animals. In particular, the choice of the *flavanone* naringenin was related to the known antioxidant and anticancer properties of this natural compound previously shown to be active in contrasting lung cancer metastasis by inhibiting migration of lung cancer cells [114,115]. In the study of Parashar et al. [48], naringenin was encapsulated in a newly designed HA-decorated chitosan–caprolacton polymeric NP. Polyanionic and hydrophobic caprolactone was used for binding naringenin. Due to its low degradation rate, this polymer has the potential to ensure a sustained release and an increased presence in the circulation of the loaded naringenin [116]. The polycationic water-soluble chitosan was added at the NP surface in order to allow the binding of the hydrophobic HA to the NP coating. Moreover, due to its mucoadhesive properties, caprolactone was used to construct NPs suitable for oral administration. Interestingly, in the in vivo experiments, oral administration of the naringenin-loaded NPs was performed both in advance, as a preventive treatment before the chemical induction of lung cancer, or at the beginning of the experiment, as a therapeutic treatment. In both conditions, the NPs showed a greater anticancer activity compared to that elicited by naringenin in its free form, especially with preventive treatment.

Instead, the isoflavon formononetin (Table 1) was encapsulated in HA-decorated NPs and bound to NPs loading docetaxel (DTX) and modified at the surface with the epidermal growth factor receptor-targeted peptide (GE11) [49]. The result was an HA and GE11 dual ligand-modified binary NP to be used for the therapy of metastatic and castration-resistant prostate cancer. Even though derived from the assembly of two different NPs, the final product size was still in the nanometric range (189.5 nm), thus allowing for easy drug delivery to tumor tissue thanks to the enhanced permeability and retention (EPR) effect [18], while it was able to induce synergistic anticancer effects if compared to the two NPs administered separately.

### 4.2. HA-Based Nanocarrier Systems Loading Non-Flavonoids

Among the studies that were recently conducted on HA-based nanoformulations delivering PheCs not belonging to the classes of flavonoids (i.e., the *non-flavonoids*) for cancer therapy, those investigating the *curcuminoid* CUR are the most prevalent (12 out of 16). Instead, among the remaining studies, one was focused on gallic acid (GA, a phenolic acid), and three on *trans*-3,5,4′-trihydroxystilbene, commonly known as resveratrol (RES, belonging to the subclass of the *stilbenes*), a natural compound having protective functions for the plants (particularly grapes) that produce it in response to fungal infections, injury, or ultraviolet irradiation [117] (Table 2).

In the first work on GA [55], HA-decorated, DOX, and GA co-delivered lipid–polymer hybrid NPs were constructed to allow a better delivery and higher antineoplastic effect of both DOX and GA in leukemia cells resistant or not to DOX. GA is an antioxidant polyhydroxyphenol shown to induce differentiation and apoptosis in leukemia cells [118,119], while DOX is a first-line cancer drug for the treatment of leukemia whose clinical use is hampered by the resistance that often arises against it. The cytotoxic effect obtained with the free DOX and GA combination was dramatically increased when the DOX–GA were co-delivered to leukemia cells in vitro encapsulated in the HA-modified nanoformulation. Correspondently, treatment with this nanoformulation inhibited the growth of tumors derived from the subcutaneous implantation of leukemia cells more than the free DOX–GA combination and prevented body weight reduction observed after the treatment with free DOX–GA.

Among the three different HA- and RES-containing nanosystems that have been recently investigated against BCa, two were HA-coated NPs [61,62], and one was an HA-based hydrogel [63]. In one case [61], the nanoformulation carried RES together with tamoxifen and was tested both on TNBC cells (CAL-51) and on estrogen and progesterone receptor-expressing BCa cells (MCF7 cells). In the other two studies [62,63], RES was the only bioactive compound to be delivered to TNBC cells (MDA-MB-231 or 4T1 cells, respectively). In the first case [61], the effect of the nanoformulation was not compared to those of free RES or free tamoxifen, but its antineoplastic action was just verified per se. This lack of adequate controls is a pitfall of the study since it does not allow us to understand if the encapsulation of RES or tamoxifen in that nanosystem could make them more active as anticancer agents and if they were acting in synergism or not. In the last two studies [62,63], independently from the nanosystem used, the HA-based and RES-enclosing nanoformulations were more efficacious than free RES in inhibiting BCa cell viability in vitro. In Shin et al.’s study [63], it was also observed that when the HA-based hydrogel was injected directly into the tumor in vivo, it increased the rate of necrosis and induced the angiogenic process more efficiently than the treatment with RES alone. The possibility of injecting this formulation directly into the tumor is worth noticing since free RES was shown to be quickly eliminated from the body [120].

The last non-flavonoid PheC we are focusing on here is the *curcuminoid* CUR (diferuloylmethane). This PheC has been recently encapsulated in HA-based nanosystems with a higher frequency with respect to all the other PheCs considered here. In fact, out of the 12 reports focusing on CUR enclosed in HA-containing nanosystems that were selected by us, 5 tested the effectiveness of CUR-loading nanosystems against tumors of the gastrointestinal tract [64,71,72,73,75]. In four reports, CUR or CUR derivatives loaded in nanosystems were tested against BCa [65,66,67,74], while in the three remaining reports, the nano-encapsulated CUR was tested against lung cancer [68], osteosarcoma [69], and OvCa [70]. The high frequency of this kind of investigation seems to be related to the large amounts of reports previously published showing the powerful anticancer effects of this natural antioxidant and dietary component [121]. However, as a free compound, CUR shows poor water solubility, fast degradation, and scarce in vivo biodistribution, thus, not allowing adequate therapeutic activity [122]. For this reason, over the last two decades, considerable effort has been expended in developing nanoformulations of CUR to make the native compound a therapeutical advanced drug [123].

The recently designed HA-containing drug-delivery systems carrying CUR or a CUR analog as cargo molecule for the therapy of BCa, were all found more effective than the compounds administered in a free form in reducing cell viability and inducing apoptosis in vitro, independently from the type of BCa (TNBC or expressing hormone receptors) [65,66,67,74]. In some cases, the nanoformulations were observed also to better reduce the in vitro cellular migration [66] or the tumor growth in vivo [66,67].

It is interesting to consider the different nature of these newly designed nanodelivery systems. In fact, among them, we identified:

(A) Polymeric micelles co-encapsulating icariin and CUR and based on pH-sensitive hydrazone bond, folic acid, and biotin-conjugated [67] (Table 2). This therapeutic strategy is interesting since it allows the codelivery of two PheCs known for their powerful anticancer activities (CUR and icariin, a prenyl-derivative of the flavonoid kaempferol). These PheCs were conjugated through a pH-sensitive hydrazone bond, which allows them to be easily released in the acidic tumor microenvironment. Moreover, to improve the recognition and internalization of the nanomicelles, besides HA, also folic acid and vitamin biotin were bound to their exterior. In fact, due to their high-grade proliferation, cancer cells need vitamins and overexpress receptors for them on their surface [124,125];
(B) HA-functionalized mesoporous silica NPs (MSNPs) enclosing CUR alone [66] (Table 2). This approach was based on a series of favorable features of MSNPs, such as their chemical and mechanical stability, the possibility to easily functionalize their surface, their suitable endocytic behavior, as well as their biocompatibility;
(C) HA- and riboflavin-coated transition metals-based nanoplatforms enclosing CUR [74] (Table 2). This interesting approach was aimed at increasing the anticancer effectiveness of transitional metals such as nickel, manganese, and iron that were used to synthesize photosensitizers for the photodynamic therapy of tumors. This effect was obtained through the inclusion of the CUR in the nanosystem to take advantage of the possible synergistic effect deriving from chemotherapy and photodynamic therapy simultaneously;
(D) HA-decorated self-assembled nanomicelles consisting of a biocompatible amphiphilic polymer formed by styrene maleic anhydride (SMA) and TPGS, loading the hydrophobic molecule of difluorobenzylidene diferuloylmethane (CDF) (a stable cytotoxic CUR analog) [65] (Table 2). Biocompatibility, specific CD44 targeting, and TPGS activity against possible drug resistance represent the strength of this nanocarrier designed for TNBC that showed the high capability to be specifically accumulated in the tumor obtained by transplanting these BCa cells in nude mice and not in a series of normal tissues investigated. The authors, however, did not investigate their effect on tumor growth in vivo.

Among the five different HA-containing nanosystems recently constructed to deliver CUR to tumors of the gastrointestinal tract, three were tested against CRC cells [64,75] and two against hepatocellular carcinoma (HCC) cells [71,73]. Overall, these nanoformulations were always more effective in reducing cancer cell viability and, in some cases, inducing apoptosis [75] or inhibiting cell migration in vitro [71], as compared to CUR administered in the free form.

CUR appears to be particularly efficient in inhibiting the growth of CRC cells, and the mechanisms underlying this powerful anticancer activity have been investigated in a recent report based on network pharmacology and molecular docking [126]. This study revealed the multi-target action of CUR and its ability to interfere with CRC cell proliferation and survival by regulating multiple signal transduction pathways. Among the HA-coated nanosystems recently constructed for encapsulating CUR and designed for CRC therapy, there are:

(A) HA cross-linked zein nanogels [64]. Zein is a natural hydrophobic biopolymer that, similarly to HA, shows high biodegradability and biocompatibility [127]. Moreover, being easily extracted from corn, it also possesses the appreciable quality of being quite inexpensive, and for all these reasons, it has attracted considerable attention as an excellent nanocarrier for hydrophobic bioactive compounds [128]. However, this was the first time that a nanogel able to carry hydrophobic drugs was created with zein and HA, starting from the hypothesis that zein could be easily cross-linked with an anion such as HA.
(B) An analogous HA–zein hydrogel NP [72] that, when compared with similar NPs where HA was substituted with other polysaccharides (arabic gum or pectin), was the most effective against CRC cells.
(C) HA–lactoferrin–EGCG-containing composite NPs [75]. The main aim of the authors was to obtain an effective anticancer CUR-loading nanosystem that could possibly be used in food and supplements for both the prevention and the therapy of CRC. For this reason, they needed to produce edible NPs, and for this purpose they used natural safe products such as lactoferrin or EGCG that could increase the stability of loaded CUR through the gastrointestinal tract, allowing its higher bioavailability. Moreover, the choice of lactoferrin and EGCG for constructing CUR-loading NPs seems very appropriate since, besides ensuring safe delivery of this bioactive compound, they could synergistically reinforce the antineoplastic effect of CUR since they are known for exerting anticancer properties against CRC cells by themselves [129,130]. Furthermore, it is very interesting that the NP decoration with HA resulted in being critical for enhancing the NP anticancer properties, especially toward CRC cells characterized by a high degree of proliferation and malignancy, such as HT-29 and CT-26 CRC cells, which are known to show increased expression of CD44 receptor. On the contrary, the anticancer effect was not obtained toward CaCo-2 cells, which are CRC cells still able to differentiate, showing low levels of CD44 expression [75].

Multiple findings have recently indicated the anticancer potential of CUR for the therapy of HCC [131,132]. In particular, several nanotechnological solutions have been investigated to overcome its scarce water solubility and bioavailability. Interestingly, recent studies verified the anticancer effects of the nanosystem against HCC cells co-cultured with stellate cells (STCs) in vitro [73,75]. This methodological approach was used with the aim of testing if it was possible to block the cross-talk between HCC and STC cells occurring in the tumor microenvironment that is considered crucial for the development and progression of hepatocarcinomas. For the same reason, in one case [73], a mixed suspension of murine HCC and murine-derived HSCs was injected in syngeneic mice, and it was observed that the newly synthesized nanosystem encapsulating CUR reduced the tumor volume more than the encapsulated bioactive products (CUR and berberine) administered alone in a free form.

Among the new HA-coated nanosystems encapsulating CUR designed for HCC therapy, there are:

(A) Liposomes decorated with HA and glycyrrhetinic acid and co-loading CUR and aprepitant [71]. This new therapeutic nanoplatform appears particularly appropriate for the therapy of HCC. In fact, it has become clear that HSCs represent important components of the tumor microenvironment, playing a crucial role in the development and progression of this kind of cancer [133]. The antiemetic drug aprepitant was found able to inhibit the activation of HSCs from their quiescent phenotype to the carcinoma-associated fibroblasts (CAFs) by blocking the neurokinin–receptor signal pathway activated by the neuropeptide SP, locally secreted by peripheral nerves [134]. Thus, the aprepitant-dependent inhibition of HSC activation could prevent the cross-talk between CAFs and HCC cells that have been involved in HCC development and progression [135]. Moreover, the co-targeting lysosomes through glycyrrhetinic acid and HA has the potential of making their delivery highly specific for this kind of cancer since, on the one hand, glycyrrhetinic acid receptors are overexpressed in HCC cells [136], and HA-specific CD44 receptors in activated HSCs [137].
(B) Analogous HA and glycyrrhetinic acid-modified liposomes synthesized by the same group [73] but co-delivering CUR and berberin, an anticancer bioactive vegetal alkaloid [138], with the identical aim of combining anti-HCS and pro-apoptotic activities and inhibiting the cross-talk between the tumor and the activated HSCs. The authors’ hypothesis assumed that berberine could have the potential to inhibit the TGF-β-induced phenotypic switch of HSCs into the fibrogenic and carcinogenic myofibroblasts [139,140].

Finally, three new HA-coated nanosystems encapsulating CUR were designed for the therapy of lung cancer [68], osteosarcoma [69], and ovarian cancer [70]. In two of these works, CUR was loaded together with the natural anticancer compound baicalein [68] and with the anticancer drug PTX [70], while in one case, it was loaded alone in the nanosystem. Overall, when CUR was encapsulated either alone or co-loaded with the other anticancer agents, it elicited higher anticancer activities both in vitro and in vivo, as compared to each of the anticancer compounds administered alone (Table 2).

The CUR-loading nanosystem constructed for the specific targeting of non-small cell lung cancer (NSCLC) was a reduction-sensitive amphiphilic carrier constituted by mannose and oligomeric HA and co-delivering also baicalein [68]. Different peculiar aspects also make this complex nanosystem particularly noteworthy. Firstly, it was designed to concomitantly target lung carcinoma cells (through the presence of oligomeric HA) and tumor-associated macrophages (TAMs), which represent a main cellular component of the tumor microenvironment (TME) involved in the promotion of tumor growth and metastasis. For this reason, NPs coating also included mannose, known for its high affinity for the TAM CD206 receptor. Moreover, in the same NP shell, HA was connected to QU (here used just for its hydrophobic properties) by using 3,3-dithiodipropionic acid (DA). Through this expedient, the easy break of the DA disulfide bonds may be obtained in the TME, where GSH is present at high concentrations, thereby leading to the specific release of the loaded bioactive compounds. Finally, the co-delivered baicalein seems a highly appropriate choice, this bioactive natural compound produced by the plant *Scutellaria baicalensis Georgi* has been demonstrated to exert multiple beneficial effects in treating a variety of lung diseases, including lung cancer [141].

Nanomicelles composed of the amphiphilic material HA–octadecanoic acid modified with alendronate were designed for loading CUR and specifically target osteosarcoma cells [69]. Alendronate, a bisphosphonate, was chosen since it possesses a marked affinity for hydroxyapatite, a mineral bone component, and, as a consequence, it may accumulate in the bone. Due to this property, it is commonly used as an effective therapeutic agent in a series of degenerative bone diseases [142]. In this study, its presence, together with HA in the polymer composing the CUR-loaded nanomicelle, made them specifically for the dual-targeting of a highly aggressive tumor of the bone, such as osteosarcoma.

The last of this group of CUR-loading nanomaterials considered here is an HA-modified NP formed by polyethylenimine (PEI) and stearic acid (SA). It was designed with the aim of improving the delivery to ovarian cancer (OvCa) of the antineoplastic drug PTX [70]. The improved synergic effect of CUR and PTX co-loaded in the nanomicelles was demonstrated both in ovarian cancer cells and in their multidrug-resistant variant. The potential that this nanosystem may have to reverse PTX resistance in ovarian cancer seems particularly worth noting. In fact, even though the currently used therapy for these ovarian cancer patients is platinum-based, in most cases, the cancer shows a tendency to develop resistance [143].

Among the drugs co-loaded with PheCs within NPs, there are: *siRNA against connective tissue growth factor (CTGF)*, which is related to drug resistance if overexpressed in triple-negative breast cancer (TNBC) [43]; *docetaxel (DTX)* [42,49]; the phototherapy agent *IR780* [47]; *doxorubicin (DOX)* [40,46,55]; *resiquimod (R848)*, an immune modulator able to activate dendritic cells present in the tumor microenvironment [45]; the conventional anticancer drug *gemcitabine (GMC)* [41], *paclitaxel (PTX)* [35,38,70], and *tamoxifen* [61]; *aprepitant (APR),* a drug used for the prevention of chemotherapy-induced nausea and vomiting that was shown to have anticancer properties and inhibit the activation of HSCs [71]; *berberine*, an isoquinoline alkaloid showing anticancer and anti-inflammatory properties [73]; the nano photosensitizer *transition metals Fe, Mn and Ni* [74].In some instances, two PheCs were co-delivered in the nanodelivery systems: *curcumin (CUR)* and *epigallocatechin-3-gallate (EGCG)* [44]; *icariin* and *CUR* [67]; *baicalin* and *CUR* [68].

## 5. Conclusions and Future Perspectives

On the basis of our critical analysis of the recent results obtained by a series of preclinical research studies that used both the natural polymer HA and one type of PheCs as simultaneous components of a single nanodelivery system, we can conclude that this represents a promising pharmacological strategy.

**Figure 2:**
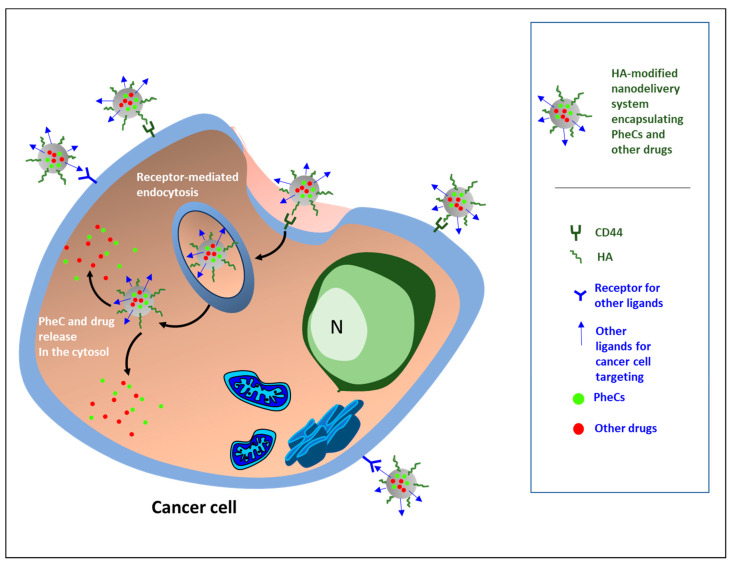
The nanoformulations designed for the targeted delivery of phenolic compounds (PheCs) to cancer cells are, in most cases, polymeric nanoparticles (NPs) having hyaluronic acid (HA) among the constituent polymers or NPs decorated at the surface with HA alone or accompanied by additional targeting ligands. Among the additional targeting ligands used, there are: *fucoidan*, that functions as a ligand for P-selectin and helps the nanosystems to be internalized in cancer cells [42,44,46]; *EGF Receptor-Targeted Peptide (GE11)* for binding to EGFR overexpressed in prostate cancer [49]; *folic acid* and *biotin* functioning as ligands for the respective receptors, both overexpressed in cancer cells [67]; *mannose* for the binding to the CD206 receptor for specifically targeting the tumor-associated macrophages (TAMs) [68]; *glycyrrhetinic acid (GA)* that binds to the specific receptor overexpressed in hepatocarcinoma cells and in activated hepatic stellate cells (HSCs) [73]; *riboflavin* [74]. Besides HA, other bioactive constituents of the nanoformulation shells are: the bisphosphonate *alendronate,* able to specifically target osteosarcoma cells [69]; *D-alpha-tocopheryl polyethylene glycol 1000 succinate (TPGS)*, able to inhibit P-gp efflux pumps, thus overcoming multidrug resistance (MDR) in cancer [40].

These newly designed nanosystems were aimed at enhancing the known anticancer activities of the PheCs themselves, as well as that of other antineoplastic drugs commonly used in the therapy of tumors, especially by enhancing their bioavailability and overcoming their poor water solubility. Moreover, these nanosystems were constructed with the additional aims of obtaining synergic anticancer effects deriving from the concomitant delivery of the drug and the PheC enclosed and also of overcoming the drug resistance and decreasing the health-threatening systemic complications usually observed with almost all anticancer therapies. In a few cases, PheCs were also enclosed in nanodelivery systems containing therapeutic agents used for radiotherapy or immunotherapy (Figure 2).

Overall, the results of the studies analyzed seem to suggest that the enhanced antineoplastic effectiveness of these nanoformulations could be related, at least in part, to the presence of HA in their external part, through the protection and specific delivery of the carried bioactive substances to cancer cells overexpressing its receptor CD44. On the other hand, the internal part encapsulated, protected, and ensured an increased release of the bioactive agents PheCs, either alone or, more often, accompanied by commonly used anticancer drugs in specific pathological locations.

Reflecting the number of positive results previously obtained on their anticancer properties, the *non-flavonoid curcuminoid* CUR was the most used for this purpose (in 12 studies in the last 5 years) among all the PheCs, followed by the *flavonoids flavonol* QU (in 6 studies) and the *flavanol* EGCG (in 6 studies), and by the *non-flavonoid stilbene* RES (in 3 studies). Finally, there exists only one study for each of the three remaining *flavonoids* recently enclosed in HA-containing nanosystems (i.e., the *flavonol* kaempherol, the *flavanone* naringenin, the *isoflavone* formononetin), as well as for the only remaining *non-flavonoid* (gallic acid, a *hydroxybenzoic acid*).

On the whole, in our opinion, the results recently obtained are encouraging and pave the way for the possible application of these nanosystems in future clinical investigations.

## Figures and Tables

**Table 1:** Hyaluronic Acid-Containing Nano-Delivery Systems Loading Phenolic Compounds (Flavonoids: Flavonols, Flavanols, Flavanones, Isoflavones) for cancer targeting.

Preclinical Cancer Model	Nanoformulation	Antineoplastic Drug/Nutraceutic Enclosed in the Same Nanoformulation or Administered Separately	PheC (*Flavonoid)* in the Nanoformulation	Anticancer Effect(s) of Nanoformulation (or Combination of Formulations) Respect to the Drugs Alone	Ref.
In vitro: A549 lung carcinoma cells	Two molecular weight HA-modified kaempferol-loaded nanostructured lipid carriers (HA-KA-NLCs). Size: HA20-KA-NLC: 92.7 ± 4.6 nm HA130-KA-NLC: 97.6 ± 4.3 nm	None	Kaempferol (*Flavonol*)	In vitro: ↓ Cell viability; ↑ IC50 ↓ Cell proliferation; ↓ Cell migration rate ↑ Apoptosis	[34]
In vitro: MCF-7/ADR human breast cancer cells and 3D spheroids In vivo: MCF-7/ADR subcutaneously injected in nude mice breast. Drugs i.v. injected when the tumor reached 200 mm^3^.	Hybrid polymeric NPs for PTX and QU co-loading. Size:187.97 ± 1.66 nm	PTX	QU (*Flavonol*)	In vitro: ↓ IC50; Apoptosis In vivo: ↓ Tumor volume	[35]
In vivo: LNCaP human prostate cancer cells transplanted in the subcutaneous space of SCID mouse dorsa. Drugs i.v. injected in tumor-bearing mice after 14 days.	A boronated derivative of HA linked to QU	None	QU (*Flavonol*)	↓ Tumor growth	[37]
In vitro: MDA-MB-231/MDR1 breast cancer cells In vivo: same cells transplanted in mice	Amphiphilic PEI-TOS/HA-QU core-shell micelles for the targeted co-delivery of PTX and QU. Size: 167.60 ± 8.185 nm	PTX	QU (*Flavonol*)	In vitro: ↓ IC50; ↑ Mitochondrial-dependent apoptosis In vivo: ↓ Tumor size	[38]
In vitro: 4T1 Breast cancer cell lines. In vivo: 4T1 Breast cancer cell lines injected subcutaneously into the right hind hip of Balb/c mice. Drugs i.v. injected every other day when the tumor reached 80–100 mm^3^	HA acid-based QU nanoformulation. Size: 235.9 ± 3.2 nm	None	QU (*Flavonol*)	In vitro: ↓ Cell viability; ↑Apoptosis In vivo: ↓ Tumor volume	[39]
In vitro: MDA-MB-231/MDR1 breast cancer cells. In vivo: MDA-MB-231/MDR1 cells inoculated subcutaneously in the breast of nude mice. Drugs i.v. injected when the tumor reached 200 mm^3^.	HA-Based Conjugate/D α-TPGS Mixed Micelles loaded with: QU (average size: 329.83 nm) or DOX (average size: 201.2 nm)	DOX	QU (*Flavonol*)	In vitro: ↓ Cell viability; ↑Apoptosis; ↓ P-gp expression In vivo: ↓ Tumor volume	[40]
In vitro: Mia-PaCa-2 and PANC-1 pancreatic cancer cell lines	HA-decorated poly-ethylene oxide NPs loaded with: CD/QU (size: 135 ± 7 nm) or GMC (size: 175 ± 10 nm)	Gemcitabine	QU (*Flavonol*)	In vitro: ↓ Cell viability; ↑ Sensitivity of cancer cells to the anti-inflammatory effect of QU loaded in NP.	[41]
In vitro: Human prostate cancer PC3 cells In vivo: PC3 cells subcutaneously implanted in SCID mice. Drugs i.v. injected 6 times at 3 day intervals	TPGS-conjugated HA and fucoidan-based NPs. 205.48 ± 8.52 nm	DXT	EGCG (*Flavanol*)	In vitro: ↓ PC3 cell viability In vivo: ↓ tumor volume and tumor weight; ↑cell apoptosis (M30 protein) and ↓ cell proliferation (Ki67 ) in tumors	[42]
In vitro: MDA-MB-231/MDR1 breast cancer cells In vivo: MDA-MB-231/MDR1 bearing Balb/c nude mice. Drugs i.v. injected 6 times at 3 day intervals.	HA-coated EGCG, siRNA and protamine nanogel Average size: 80 nm	siRNA (for silencing CTGF, associated to drug resistance, promotion of cell proliferation, and migration)	EGCG (*Flavanol*)	In vitro:↓ MDA-MB-231/MDR1 cell viability and ↑apoptosis; ↓ expression of the drug resistance associated proteins cIAP1, Bcl-xL, and CTGF In vivo: ↓ Tumor volume	[43]
In vitro: Luc PC3 prostate cancer cells In vivo: Luc PC3 cells orthotopically injected in SCID mice. Drugs i.v. injected 6 times at 3 day intervals	HA, fucoidan, and poly(ethylene glycol)-gelatin NPs encapsulating EGCG and CU. Size: 197.73 ± 18.53 nm	CU	EGCG (*Flavanol*)	In vitro: ↓ PC3 cell viability; In vivo: ↓ Tumor cell proliferation (Ki-67)	[44]
In vitro: B16F10 mouse melanoma cells and DC2.4 mouse dendritic cell line. In vivo: B16F10 cells subcutaneously injected into the flank of C57BL/6J mice. Drugs i.v. injected every 3 days for four times.	Nanogels containing HA, cyclodextrin, and pH-sensitive ketone cross-linker DMAEP coloading ECGC and R848. Size: from 161.4 ± 2.86 to 170.17 ± 4.95 nm	Resiquimod (R848) (immune modulator)	EGCG (*Flavanol*)	In vitro: ↑ Maturation of dendritic cells and CTL stimulation. In vivo: ↑ PDL1 expression in tumors; ↓ CTL activation and infiltration into tumors; ↑ Treg suppressive effects	[45]
In vitro: Stable luciferase-expressing human MKN45 gastric cancer cells (Luc MKN45); In vivo: Luc MKN45 cells orthotopically inoculated in SCID mice. Drugs injected 5 times within 2 weeks	Fucoidan and TPGS-conjugated HA-based NPs. Size: 200–230 nm	DOX	EGCG (*Flavanol*)	In vitro: ↓ PC3 cell viability; arrest in G2/M cell cycle phase; ↑ apoptosis: In vivo: ↓ luminescence in luciferase-expressing gastric tumors; ↑cell apoptosis (M30 protein) and ↓cell proliferation (Ki67) in tumors	[46]
In vitro: A549 lung carcinoma cells. In vivo: A549 cells microinjected into the yolk of zebrafish embryos cultured for 48 h. Embryos treated with the drugs after 4 h.	Mitochondrial-targeting reduction-responsive nano drug delivery system (EGCG@THSI NPs). Size: about 173.2 nm	IR780-iodide (IR780) (photoactivator)	EGCG (*Flavanol*)	↓ Cell proliferation (in vitro and in vivo) ↑ ROS production ↓ Cell invasion, metastasis and angiogenesis (in vivo)	[47]
In vitro: A549 lung carcinoma cells. In vivo: chemically-induced lung cancer in Wistar rats by 3 i.p. injections of urethane, every 2 days for a week. Drug orally administrated (50 mg/kg) at the start of the experiment or 15 days prior the beginning of the experiment.	HA-decorated caprolactone NPs. Size: 251.6 ± 3.22 nm	None	Naringenin (*Flavanone*)	In vitro: ↓ A549 cell viability; ↑ Cell-cycle arrest in G2-M phase In vivo: ↑ Animal survival ↓ Lung weight	[48]
In vitro: Human PC3 prostate adenocarcinoma cell line. In vivo: PC3 cell subcutaneously injected into the right flank of Balb/c nude mice. Drugs i.v. injected as the tumor reached 100 mm^3^.	GE11-modified nanoparticles (GE-NPs) loaded with DTX assembled with HA-decorated NPs (HANPs) encapsulating Formononetin Size: 189.5 ± 3.3 nm,	DTX	Formononetin (*Isoflavon*)	In vitro:↓ PC3 cell viability In vivo: ↓ Tumor volume ↑ Drug distribution in tumor	[49]

CD/QU: 2-hydroxypropyl-β-cyclodextrin/Quercetin; CTGF: connective tissue growth factor; CU: curcumin; DOX: doxorubicin; DXT: docetaxel; EGCG: epigallocatechin-3-gallate; HA: hyaluronic acid; KA: kaempferol; MDR: multidrug resistance; NP: nanoparticles; PEI-TOS/HA-QU: Polyethyleneimine-Tocopherol Hydrogen Succinate/Hyaluronic Acid-Quercetin; PTX: paclitaxel; QU: Quercetin; TPGS: D-Alpha-tocopheryl polyethylene glycol 1000 succinate.

**Table 2:** Drug Nano-Delivery Systems Including Hyaluronic Acid and Phenolic Compounds (Non-Flavonoids: Phenolic acids, Stilbenes, Curcuminoids) for cancer targeting.

Preclinical Model	Nanoformulation (as Reported by the Authors)	Antineoplastic Drug/Nutraceutic enclosed in One Nanoformulation or Administered Separately	PheC *(Non-Flavonoid)* in the Nanoformulation	Anticancer Effect(s) of the Nanoformulation (or Combination of Formulations) Respect to the Enclosed Free Compounds	Ref.
In vitro: DOX-resistant HL-60 promyelocytic leukemia cells and DOX-resistant K562 chronic myeloid leukemia cells. In vivo: DOX-resistant HL-60 cells subcutaneously injected in Balb/c nude mice. Drugs i.v. injected every 3 days for 7 times.	Lipid-polymeric hybrid NPs consisting in: HA conjugated with PEG-DSPE co-loading DOX and GA. Size: 165.7 ± 4.6 nm	DOX	GA (*Phenolic acid*)	In vitro: ↓Cell viability ↓ IC50 (maximally at 2:1 DOX/GA ratio) In vivo: ↓ Tumor volume ↓ Decrease in body weight	[55]
In vitro: Human MCF-7 and CAL-51 breast cancer cell lines.	Liquid crystalline NPs (LCNPs) enclosing Tamoxifen plus RES and coated with multiple layers of chitosan and HA. Size: about 217 nm	TAM	RES *(Stilbene)*	In vitro: ↓ Cell viability *; ↑ Apoptosis * In vivo: No change in body weight during treatment	[61]
In vitro: 4T1 murine breast cancer cells	RES-loaded Zein-SHA NPs. Average size: about 152.13 nm	None	RES *(Stilbene)*	In vitro: ↓ Cell viability ↓ IC50	[62]
In vitro: MDA-MB-231 triple-negative breast cancer cells In vivo: subcutaneous inoculation of MDA-MB-231 cells in the abdomen of nude mice. Drugs injected into the tumors as they reached a volume of about 80 mm^3^.	Injectable Res-Cx-HA hydrogel Size: Non reported	None	Resveratrol (RES) *(Stilbene)*	In vitro: ↓ Cell viability; In vivo: Tumor tissue inject with the Hydrogel: ↑ necrosis rate of tumor tissue; ↓ Angiogenesis	[63]
In vitro: CT26 colon cancer cell line; In vivo: CT26 cells xenografted in the flank of Balb/c nude mice. Drugs i.v. injected every day for 2 weeks.	HA-Zein-CUR NG Size: from 200–250 nm.	None	CUR *(Curcuminoid)*	In vitro: ↓ Cell viability; ↑ Apoptosis In vivo: ↓ Tumor volume and weight	[64]
In vitro: MDA-MB-231 and MDA-MB-468 breast cancer cells	CDF loaded in HA-SMA-TPGS nanomicelles. Average size: 129.4 nm	None	CDF *(Curcuminoid)*	In vitro: ↓ Cell viability; ↑ Apoptosis ↑ PTEN (pro-apoptotic) and ↓ NF-kB (tumorigenic) expression	[65]
In vitro: MDA-MB-231 breast cancer cell line. In vivo: Swiss albino mice injected with Erlich Ascites Carcinoma. Drugs i.v. injected after 10 days every 2 days for 2 weeks.	HA-tagged mesoporous silica NPs loaded with CUR. Average size: 161.3 nm	None	CUR *(Curcuminoid)*	In vitro: ↓ Cell viability; ↓ in-vitro cellular migration; ↑ Apoptosis; Cell cycle arrest at G2/M phase In vivo: ↓ Tumor volume and tumor mass	[66]
In vitro: MCF-7 cells breast cancer cells and breast cancer stem cells. In vivo: MCF-7 cells injected in nude mice. Drugs i.v. injected as tumors reached 400 mm^3^	Icariin and CUR co-encapsulated in polymeric micelles based on pH-sensitive hydrazone bond, FA and biotin-conjugated HA. Size: 162.7 ± 5 nm.	Icariin	CUR *(Curcuminoid)*	In vitro: ↓ Cell viability; ↓ Invasion ability (Transwell assay) In vivo: ↓ Tumor volume	[67]
In vitro: A549 lung carcinoma cells and RAW264.7 cells. In vivo: A549 cells trasplanted in mice. Drugs i.v. injected as the tumor reached an appropriate size.	QU-dithiodipropionic acid-oligomeric HA-mannose-ferulic acid self-assembled and encapsulating CUR and Baicalin. Size: 121.0 ± 15 nm	Baicalin	CUR *(Curcuminoid)*	In vitro: ↓ Cell viability; In vivo: ↓ Tumor volume; ↓ Decrease in body weight	[68]
In vitro: MG-63 osteosarcoma cells In vivo: MG-63 cells injected into the right tibia of nude mice; at 12 days, drugs injected every 2 days for 20 days.	ALN-HA-C18 loading CUR. Size: 118 ± 3.6 nm	None	CUR *(Curcuminoid)*	In vitro: ↓ Cell viability; In vivo: ↓ Tumor volume;	[69]
In vitro: Human SKOV3 and SKOV3-TR30 ovarian cancer cells (multi-drug resistant); In vivo: SKOV3 cells subcutaneously xenografted. Drugs i.v. injected as tumor reached 200 mm^3^.	HA-coated PEI-SA copolymer co-encapsulating PTX and CUR. Average Size: 187.77 nm	PTX	CUR *(Curcuminoid)*	In vitro: ↓ IC50 (in both the cells) ↓ Cell invasiveness (of both cells) In vivo: ↓ Tumor volume	[70]
In vitro: LX-2 human hepatic stellate cells (HSCs) and SMMC-7721 human hepatocarcinoma cells (HCCs). In vivo: H22 mouse HCCs, mouse HSCs and SP subcutaneously injected into the right flank of mice. Drugs i.v. injected 7 times as tumors reached 150 mm^3^.	HA and glycyrrhetinic acid-modified liposomes co-delivering aprepitant and CUR. Size: 117.40 ± 0.62	APR	CUR *(Curcuminoid)*	In vitro: ↓ Cell viability of LX-2 cells and of SP-treated LX-2 + HCCs co-culture; ↓ Cell migration in HCCs and in SP-treated LX-2 + HCCs co-culture In vivo: ↓ Tumor volume	[71]
In vitro: HCT116, HCT8, and HT29 colon cancer cells.	Zein-HA NPs enclosing CUR. Average size: about 300 nm	None	CUR *(Curcuminoid)*	In vitro: ↓ Cell viability of all cancer cell and IC50 with Zein-HA NPs (respect to CUR alone or to all other NPs)	[72]
In vitro: Human HCCs (BEL-7402), human-derived HSCs (LX-2), mouse HCCs (H22), and mouse HSCs (mHSCs); In vivo: H22+mHSCs injected in the flank of Balb/c mice. Drugs i.v. injected as tumors reached 200 mm^3^.	Glycyrrhetinic acid- and HA-modified liposomes co-delivering Berberine and CUR. Size: 159.39 ± 3.16 nm	Berberine	CUR *(Curcuminoid)*	In vitro: ↓ Cell viability of BEL-7402 cells and co-cultured BEL-7402+LX- cells; In vivo: ↓Tumor volume in H22+m-HSC tumor-bearing mice model	[73]
In vitro: Human breast adenocarcinoma cell line (MDA).	HA- and riboflavin-coated transition metals-based nanoplatforms enclosing CUR. Average size: about 70 nm	None	CUR *(Curcuminoid)*	In vitro: ↓ Cancer cell viability (vs. NPs not containing CUR, not vs. CUR alone)	[74]
In vitro: human HT-29 and mouse CT-26 colon cancer cells	Lactoferrin-EGCG-NP coated with HA and loading CUR. Average size: 144.7 nm	None	CUR *(Curcuminoid)*	↓ Cancer cell viability ↑ Apoptosis	[75]

ALN-HA-C18: Alendronate-HA-octadecanoic acid; APR: aprepitant; CD/QU: 2-hydroxypropyl-β-cyclodextrin/Quercetin; CDF: 3,4-difluorobenzylidene diferuloylmethane; CUR: curcumin; DMAEP: 2,2-dimethacroyloxy-1-ethoxypropane; EGCG: epigallocatechin-3-gallate; FA: folic acid; GA: gallic acid; HA: hyaluronic acid; HA-Zein-CUR NG: HA cross-linked zein nanogel including curcumin; HCD-A: 2-hydroxypropyl-β-cyclodextrin acrylate; MDR: multidrug resistant; ND: not detected; NPs: nanoparticles; PEG-DSPE: polyethylene glycol-distearoyl phosphoethanolamine; PEI-SA: polyethylenimine and stearic acid; PTX: paclitaxel; QU: Quercetin; RES: resveratrol; Res-Cx-HA: RES-loaded click-crosslinked HA; SHA: low-molecular-weight sodium hyaluronate); SMA: styrene maleic anhydrase polymer; SP: substance P; TAM: tamoxifen; TPGS: Tocopherol polyethylene glycol 1000 succinate. *: In this case, the comparison was made only between cells treated or not with the nanosystem.

## Data Availability

Not applicable.
